# Role of Mitochondria in the Redox Signaling Network and Its Outcomes in High Impact Inflammatory Syndromes

**DOI:** 10.3389/fendo.2020.568305

**Published:** 2020-09-23

**Authors:** Natalia D. Magnani, Timoteo Marchini, Valeria Calabró, Silvia Alvarez, Pablo Evelson

**Affiliations:** ^1^Universidad de Buenos Aires, Facultad de Farmacia y Bioquímica, Departamento de Química Analítica y Fisicoquímica, Cátedra de Química General e Inorgánica, Buenos Aires, Argentina; ^2^Universidad de Buenos, CONICET, Instituto de Bioquímica y Medicina Molecular (IBIMOL), Facultad de Farmacia y Bioquímica, Buenos Aires, Argentina; ^3^Universidad de Buenos Aires, Facultad de Farmacia y Bioquímica, Departamento de Química Analítica y Fisicoquímica, Cátedra de Fisicoquímica, Buenos Aires, Argentina

**Keywords:** mitochodria, inflammation, oxidative stress, environmental pollution, reactive oxygen species

## Abstract

Inflammation is associated with the release of soluble mediators that drive cellular activation and migration of inflammatory leukocytes to the site of injury, together with endothelial expression of adhesion molecules, and increased vascular permeability. It is a stepwise tightly regulated process that has been evolved to cope with a wide range of different inflammatory stimuli. However, under certain physiopathological conditions, the inflammatory response overwhelms local regulatory mechanisms and leads to systemic inflammation that, in turn, might affect metabolism in distant tissues and organs. In this sense, as mitochondria are able to perceive signals of inflammation is one of the first organelles to be affected by a dysregulation in the systemic inflammatory response, it has been associated with the progression of the physiopathological mechanisms. Mitochondria are also an important source of ROS (reactive oxygen species) within most mammalian cells and are therefore highly involved in oxidative stress. ROS production might contribute to mitochondrial damage in a range of pathologies and is also important in a complex redox signaling network from the organelle to the rest of the cell. Therefore, a role for ROS generated by mitochondria in regulating inflammatory signaling was postulated and mitochondria have been implicated in multiple aspects of the inflammatory response. An inflammatory condition that affects mitochondrial function in different organs is the exposure to air particulate matter (PM). Both after acute and chronic pollutants exposure, PM uptake by alveolar macrophages have been described to induce local cell activation and recruitment, cytokine release, and pulmonary inflammation. Afterwards, inflammatory mediators have been shown to be able to reach the bloodstream and induce a systemic response that affects metabolism in distant organs different from the lung. In this proinflammatory environment, impaired mitochondrial function that leads to bioenergetic dysfunction and enhanced production of oxidants have been shown to affect tissue homeostasis and organ function. In the present review, we aim to discuss the latest insights into the cellular and molecular mechanisms that link systemic inflammation and mitochondrial dysfunction in different organs, taking the exposure to air pollutants as a case model.

## Introduction

Mitochondria have been historically identified as the main source of cellular energy, by coupling the oxidation of fatty acids and pyruvate with the production of adenosine triphosphate (ATP) by the electron transport chain (1, 2). They are complex organelles that play a wide range of functions, including regulation of Ca^2+^ homeostasis, apoptosis, and differentiation (3, 4). Mitochondria are also an important source of ROS (reactive oxygen species) within most mammalian cells and are therefore highly involved in oxidative stress, where increased ROS production might contribute to mitochondrial damage in a range of pathologies (5, 6). They also play a significant role in a redox signaling system where an interplay is displayed from the organelle to the rest of the cell. Recently, new functions for the mitochondria were proposed, particularly linking the alterations in the mechanisms linked to ROS generation with the inflammatory responses involved in different pathological conditions (7–9). In this review, we will discuss the role of the mitochondria in the cellular and molecular mechanisms that link systemic inflammation and mitochondrial dysfunction in different organs, taking the exposure to air pollutants as a case model.

### Reactive Oxygen Species and Oxidative Stress

The concept of ROS was originally presented to describe the luminol chemiluminescence of activated human monocytes in 1982 (10). The concept was rapidly adopted by the scientific community, despite the fact that the cited work did not include the definition of the involved chemical species. Originally, ROS comprised superoxide anion (${\text{O}}_{2}^{•-}$), hydrogen peroxide (H_2_O_2_) and hydroxyl radical (HO^•^), which derive from the partial reduction of molecular O_2_ (11, 12). When molecular O_2_ in its basal state accepts an electron, ${\text{O}}_{2}^{•-}$ will be the product obtained, a reactive chemical species with only one unpaired electron. Adding a second electron will lead to the formation of the peroxide ion (${\text{O}}_{2}^{2-}$), from which H_2_O_2_ is its common form at physiological pH (13, 14). Since H_2_O_2_ does not have unpaired electrons is less reactive. However, this molecule is considered a reactive O_2_ species because the O-O bond is relatively weak (bond energy: 138 kJ/mol). Therefore, it can decompose leading to HO^•^ formation, whose reactivity is so high that it reacts very close to its site of formation (14, 15).

In biological systems, the reaction ${\text{O}}_{2}^{•-}$ with H_2_O_2_ in the presence of transition metals such as Fe or Cu leads to the formation of HO^•^ in a reaction postulated by F. Haber and J. Weiss and known as the Haber-Weiss reaction (16):

(1) $$\begin{array}{l} {\text{O}}_{\text{2}}^{•-}{\text{+H}}_{\text{2}}{\text{O}}_{\text{2}}\overset{{\text{Fe}}^{\text{2+}}}{\to }{\text{O}}_{\text{2}}{\text{+OH}}^{-}\text{+}\;{\text{HO}}^{•} \end{array}$$

This reaction proceeds in two consecutive steps: first, Fe^3+^ is reduced by the action of O_2_,

(2) $$\begin{array}{l} \text{F}{\text{e}}^{3+}+{\text{O}}_{2}^{•-}\to \text{F}{\text{e}}^{2+}+{\text{O}}_{2} \end{array}$$

while in the second step H_2_O_2_ reacts with Fe^2+^ to produce hydroxyl radical, also known as the Fenton reaction.

(3) $$\begin{array}{l} \text{F}{\text{e}}^{2+}+{\text{H}}_{2}{\text{O}}_{2}\to \text{F}{\text{e}}^{3+}+\text{O}{\text{H}}^{-}+\text{H}{\text{O}}^{•} \end{array}$$

The Fenton and Haber-Weiss reactions are responsible for the generation of HO^•^ in biological systems and therefore are involved in the pathophysiological mechanisms of diseases where oxidative stress plays a significant role (17).

The concept of ROS was later extended and today it is accepted that these chemical species comprises ${\text{O}}_{2}^{•-}$, H_2_O_2_, and HO^•^, some intermediates of the free radical-mediated lipid peroxidation, such as peroxyl radical(ROO•) and singlet oxygen (^1^O_2_) and also organic peroxides (ROOH) and peroxynitrite (ONOO^−^), the latter product of the reaction between and ${\text{O}}_{2}^{•-}$ and nitric oxide (NO) (18, 19).

ROS are generated during normal intracellular metabolism in mitochondria and peroxisomes, as well as from a variety of cytosolic enzyme systems.

A complex antioxidant defense system comprising antioxidant enzymes and low molecular mass reductants counteracts and regulates overall ROS at physiological levels (20). The antioxidant enzymes act as a coordinated system and includes superoxide dismutase, catalase, glutathione peroxidase and peroxiredoxins; each one of them comprised by several isoforms with specific substrates and cellular locations (21, 22). Low molecular weight antioxidants are represented by glutathione, tocopherols, ascorbic acid, and carotenoids (23).

When an increased rate of ROS production occurs and the antioxidant system is overwhelmed, a disruption in the redox balance is observed and an oxidative stress situation can be characterized. At first, the adverse effects produced by increased ROS levels were believed to result in oxidative damage to proteins, lipids, and DNA. However, in addition to these effects, the increase over physiological ROS levels may also trigger diverse stress signals that can activate specific redox-sensitive signaling pathways. Once activated, the signaling pathways may have either deleterious effects or potentially adaptive functions (24, 25).

The definition of oxidative stress was first introduced by Sies (26) and recently updated to include the role of redox signaling. It is defined as an imbalance between oxidants and antioxidants in favor of the oxidants, leading to a disruption of redox signaling and control and/or molecular damage (27, 28). Based in this new description and given the enormous variety and range of ROS and related oxidant and antioxidant compounds, efforts were made to introduce a different scale that distinguishes basal (physiological) oxidative stress from a situation where cytotoxic responses are observed (29).

**Figure 1:**
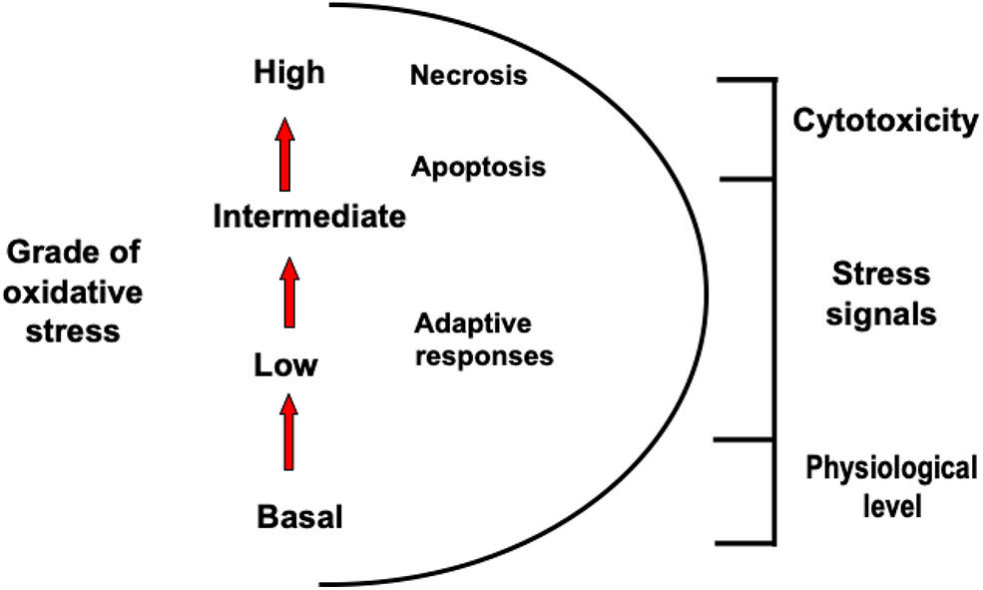
Different types of oxidative stress. Oxidative stress can be classified into different grades that are related to specific responses and cellular outcomes.

Figure 1 shows how intensity of oxidative stress can be classified into different grades that are related to specific responses and cellular outcomes. The different intensity response to oxidative stress allows biological systems to adequately react to these challenges in a dose-dependent manner (28).

### Mitochondria and NADPH Oxidases as Relevant Sources of ROS

In every eukaryotic cell ROS are generated under normal physiological conditions through enzymatic auto-oxidation reactions, which can involve both endogenous and xenobiotic compounds. The production sources can be localized at various specific subcellular systems such as plasma membrane, cytosol, peroxisomes, or organelle's membranes. Here we will be focused on NADPH oxidases (NOX) (30) and the mitochondrial electron transport chain (31) as they can be considered the predominant production sources in several pathophysiological situations.

Mitochondria are the main responsible for the cellular ATP synthesis by oxidative phosphorylation, as 36 ATP molecules are generated for each glucose molecule that is oxidized, as opposed to the two ATP molecules generated by glycolysis in the cytoplasm. It is based on transfer of electrons through the mitochondrial respiratory chain comprised of a series of integral proteins located on the inner membrane physically and functionally grouped into complexes (complex I, NADH dehydrogenase; complex II, succinate dehydrogenase; ubiquinol-cytochrome c oxidoreductase complex III; and complex IV, cytochrome oxidase), allowing a sequential arrangement that facilitates the transfer of electrons between them, determining a high speed and efficiency of the system. The driving force that governs the electron transfer along the complexes is the standard reduction potential of each complex, which depends on the concentrations of the oxidized and reduced forms of each one at a certain pH and ends at the O_2_ as the last electron acceptor, which is reduced to H_2_O. This process leads to protons (H^+^) pump from the matrix to the intermembrane space generating the proton motive force across the inner membrane. It is now known that not only ATP synthesis, but ROS production as well is a biological mitochondrial function, due to their potential key role in cell signaling to support mitochondrial integrity and adaptive responses that control homeostasis and promote health span (32–35). Electrons can leak to O_2_ creating ${\text{O}}_{2}^{•-}$ in different respiratory chain sites as complex I and III, the major site of cellular O_2_ consumption (36–38). In complex I, ${\text{O}}_{2}^{•-}$ is produced through the electron transfer to O_2_ during the NADH-dehydrogenase flavin mononucleotide semiquinone autoxidation, while in complex III the electron is transferred by the ubiquinone. Under normal conditions complex I contributes to one third and complex III to two thirds of mitochondrial ${\text{O}}_{2}^{•-}$ production. Complex II has also been suggested as a mitochondrial ${\text{O}}_{2}^{•-}$ production source through the reverse electron transfer, that can be regulated by ATP-sensitive potassium channels and mitochondrial ATP level (38, 39). Mitochondrial ${\text{O}}_{2}^{•-}$ may also be produced by α-ketoglutarate dehydrogenase, pyruvate dehydrogenase, glycerol-3-phosphate dehydrogenase, fatty acid β-oxidation. Under pathophysiological conditions, changes in bioenergetic states may result in mitochondrial substrate availability alterations, affecting the mitochondrial ROS production source (38, 40). The highly reactive ${\text{O}}_{2}^{•-}$ is considered as the stoichiometric precursor of mitochondrial H_2_O_2_ production as ${\text{O}}_{2}^{•-}$ produce mainly on the matrix side is rapidly dismutated to H_2_O_2_ by mitochondrial manganese SOD (MnSOD). H_2_O_2_ is a neutral molecule that can easily diffuse through mitochondrial membranes regardless of the organelle energization. Given the variety of mitochondrial ROS and the bioenergetic conditions requirements, mitochondria are part of a dynamic networks within the cell that involves mitochondrial fission and fusion processes and subcellular trafficking in order to control subcellular location of ATP or ROS release to support specific cell functions (12, 41–43).

The NOX represent a family of enzymes whose function is to mediate regulated cellular production of ROS by transferring electrons from NADPH, to reduce O_2_ to ${\text{O}}_{2}^{•-}$. NOX enzymes participate in important biological and pathophysiological processes. Although it is considered the defense against pathogens as the major NOX function, they also participate in inflammation response, cell signaling and regulation of cell growth, differentiation, and death (43). During inflammation NOX complex gets assembled and activated within the phagosomes to generate intraphagosomal ROS in order to kill ingested microorganismos by oxidative mechanisms (43–46). Up to now, seven NOX isoforms have been described: NOX1, NOX2, NOX3, NOX4, NOX5, and two higher molecular weight counterparts called dual oxidases, DUOX 1 and DUOX 2 (47, 48). Despite the fact that all isoforms structure shares the main functional domains, each NOX presents a difference in its regulation, activation and subcellular location. Regarding subcellular localization, different NOX catalytic and regulatory subunits have been detected in diverse cellular membranes and intracellular structures. Isoform NOX1, NOX2, NOX4, and NOX5 have been frequently informed to be found at the plasma membrane (45, 49–51). According to the reports, for NOX4, a constitutively active isoform enzyme, has been detected in the endoplasmic reticulum (52) as well as uniquely localizes to the mitochondria in various endothelial cell types (49, 53–55). Moreover, NOX isoforms also release different oxidant species. For example, NOX1 and NOX2 generate ${\text{O}}_{2}^{•-}$ (43, 56), while NOX4 is responsible for the basal production of H_2_O_2_ (57), and NOX5 produces H_2_O_2_ as well but in a Ca^2+^-dependent fashion (58). The increased NOX activity that leads to an augmented production of oxidizing species such as ${\text{O}}_{2}^{•-}$ and consequently H_2_O_2_, has been largely associated with various pathological situations (59).

Regarding signaling functions, when ROS are released from NOX, in a regulated and deliberate fashion, they are able to activate tyrosine phosphatase. This enzyme is involved in numerous transcription factors phosphorylation required to modulate different cell proliferation, differentiation, or death pathways (39). All members of the NOX family are multi-transmembrane proteins containing a flavocytochrome b558 (gp91 phox) that is associated with another transmembrane protein, p22phox. The gp91phox subunit contains the binding sites for NADPH and FAD and 2 heme groups necessary for transmembrane electrons transport from NADPH to O_2_, generating ${\text{O}}_{2}^{•-}$. Activation of the enzyme depends on the phosphorylation of 3 cytosolic regulatory proteins (p47phox, p67phox, p40phox) and together with GTPase and Rac1 they assemble to the transmembrane domains forming the functional NOX. In this sense, the enzyme goes from being at rest to being quickly activated against different cellular stimuli (47).

### Redox Signaling and Crosstalk Between ROS Sources

Under normal physiological conditions, production of ROS is not only down-regulated by several mechanisms, but also highly restricted to specific subcellular sites (12, 39, 60). Compartmentalization of ROS production within cells is important not only in terms of target specificity and selectivity but also in elicit redox signaling or oxidative damage. However, when cells experience pathophysiological situations, an excessive ROS production that overwhelms the antioxidant defense systems, results in cellular dysfunction due to oxidative stress. The cellular redox status has been assessed through different approaches like measurements of GSH status, lipid, proteins and DNA oxidation showing these outcomes association with the development of several diseases such as atherosclerosis, heart failure, hypertension, ischemia/reperfusion injury, cancer, aging, and neurodegeneration (39, 43, 61).

An interplay between specific ROS sources has been recognized, where the consequences of the redox crosstalk, mainly between mitochondria and NOX, is of increased interest in the last years. In this scenario, various ROS sources interaction stimulates each other in a positive feedback fashion, resulting in a complex oxidative stress and redox signaling network (39, 62, 63). It is well-documented that all main ROS sources are, at the same time, regulated by oxidation. ROS production sources enhancement was first described for mitochondria-to-mitochondria communication, where ROS released from one organelle triggers ROS production by another organelle. Oxidative damage of the mitochondrial respiratory chain constituents leads to mild uncoupling resulting in an augmented ROS production (62). Also, inactivation of MnSOD due to oxidation or nitration, increase cytosolic ${\text{O}}_{2}^{•-}$ levels (64). Therefore, mitochondria are efficient ROS amplifiers that may further feed this vicious cycle. Once ROS within mitochondria reach certain stationary state levels, the organelle is able to display specific mechanisms in order to interact with other mitochondria or ROS sources. Two different mechanisms were proposed to mediate the mitochondrial ROS production enhancement (40). In the first one, an increase of the mitochondrial respiratory chain- ROS release activates the mitochondrial permeability transition pore (mPTP) causing depolarization of the inner and outer membranes, which in turn yields a burst of ROS released to the cytosol (31). A second mechanism involves the direct opening of an inner mitochondrial membrane anion channel allowing ROS to enter the intermembrane space and then released into the cytosol via the voltage-dependent anion channel (40).

The mitochondrial ROS-induced ROS release concept was then widened to communication between different ROS sources like the described mitochondria to NOX crosstalk (63). In recent years, those mechanisms have been observed in different experimental models, namely aging, in response to nitroglycerin therapy, MnSOD deficiency, by angiotensin II, hypoxia or sepsis among others (30, 63, 65, 66). The link can either be triggered by NOX-released ROS to mitochondria or vice versa from mitochondrial site to NOX level, depending on the pathogenesis of the above-mentioned diseases. In both pathways, the opening of the mitochondrial ATP-sensitive potassium channels (mitoK_ATP_) seems to play an important role and has been tested through the use of inhibitors or channel openers (63). It has been shown mitoK_ATP_ becomes activated by NOX-released ROS (67). Opening of the mitoK_ATP_ stimulates the potassium influx that shifts the mitochondrial matrix to an alkalization, initiating swelling, mild mitochondrial uncoupling, and ROS production. The opening of mitoK_ATP_, triggers changes in the mitochondrial membrane potential, opening the mitochondrial permeability transition pore (mPTP) which leads to a subsequent mitochondrial ROS release to the cytosol resulting in additional NOX activation in a vicious circle (68). In a mechanism similar to the mitochondria to mitochondria interplay, once mPTP and mitoK_ATP_ channels are opened, mitochondrial ROS release into the cytoplasm activates protein kinase C (PKC) leading to NOX ensemble. This interaction was confirmed by inhibition of NOX enzymes, which prevented, for example, the mitochondrial dysfunction induced by angiotensin II (39, 63).

**Figure 2:**
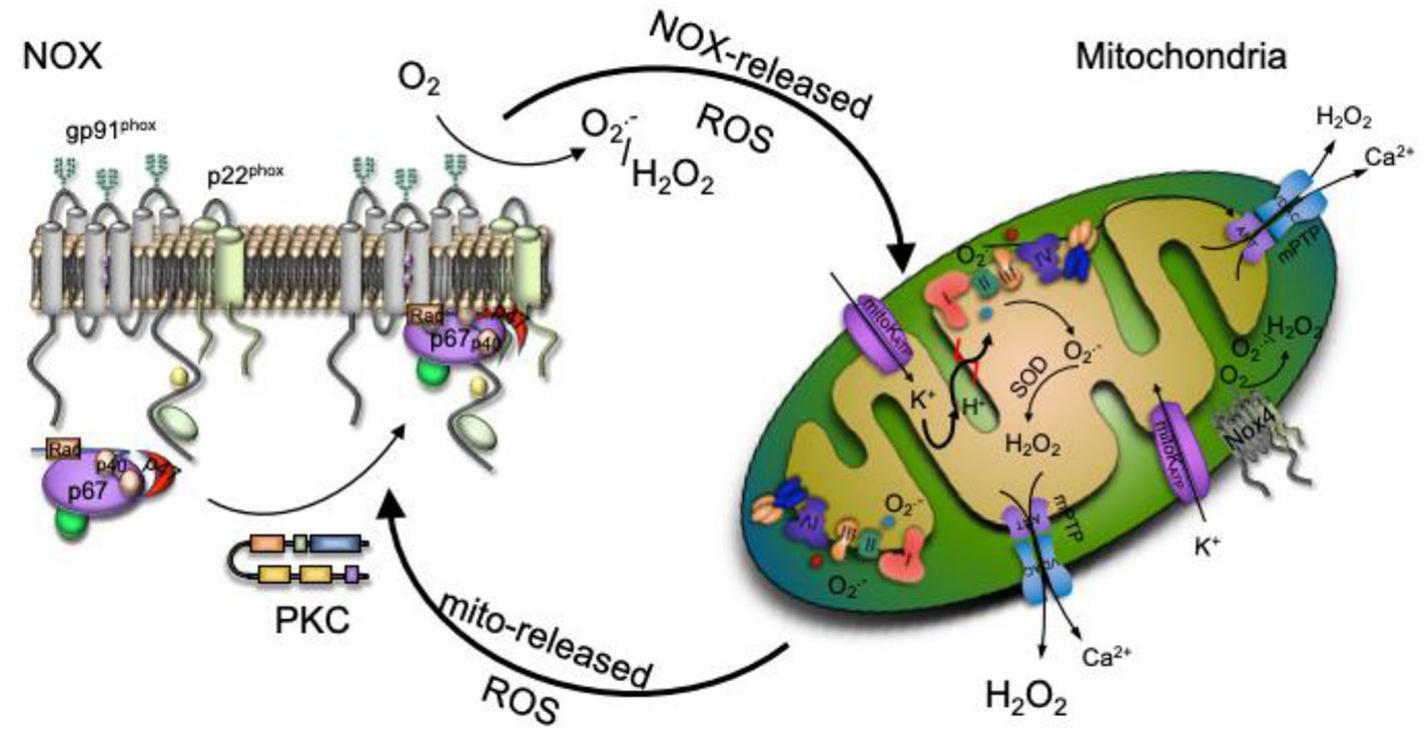
Redox signaling could be enhanced by the interplay between ROS production main sources. Increased production of NADPH oxidase (NOX)-derived ROS activate the ATP-sensitive potassium channel (mitoK_ATP_ channel), causing a matrix alkalinization through a K^+^ influx, leading to mild uncoupling and an increase mitochondrial respiratory chain- ROS production that activates the mitochondrial permeability transition pore (mPTP). The escape of mitochondrial ROS into the cytosol due to the mPTP opening activates protein kinase C (PKC), which phosphorylates the NOX cytosolic unit triggering NOX ensemble and activation.

Given the complex cellular redox network depicted in Figure 2, better knowledge about the main ROS sources crosstalk potential mechanisms, along with understanding of the switch from redox signaling to oxidative damage will help in the searching for new therapeutic approaches and the development of more target specific antioxidants.

### Inflammation and the Role of Mitochondria

Inflammation is a physiological response of the host against infection or tissue injury that occurs in order to eliminate the threat and restore tissue homeostasis, thus resolving the injury/infection and preventing damage progression (69–71). When the innate immune system detects tissue damage or senses a “danger” signal, activates the first line of defense and repair programs which initiate the inflammatory response triggering various mechanisms (72). The inflammation process is induced by a wide range of inflammatory stimuli, such usinfection, trauma, autoimmune disorders, ischemia, chemical, toxin exposure, among others, and is recognized by a combination of three classical clinical signs: hyperthermia, vasodilation and edema. Despite the cause of damage, harmful stimuli initially activate an acute inflammatory response where tissue-resident macrophages play a key role, not only detecting the infection or injury but also influencing normal tissue homeostasis. This inflammatory response is initially localized at the site of injury and includes distinct cell types, such as neutrophils and macrophages, and leads to the production and release of a variety of soluble inflammatory mediators that will act on tissues and organs affecting their functionality and metabolic state (69, 72–74). These mediators include cytokines and chemokines, that serve to amplify the local response, driving cellular activation and migration of additional inflammatory cells to the damage site. Cytokines can also modulate the function of proximate cells, including secretion of more pro-inflammatory cytokines with synergistic effects (75, 76).

Also, these mediators change vascular permeability and endothelial function, increasing endothelial expression of members of the selectin family of adhesion molecules (L-selectin, E-selectin), that allow the rolling along the vascular endothelium, and the activation of integrins that bind to endothelial vascular adhesion molecules (ICAM-1 and VCAM-1) facilitating the immobilization and transmigration of leukocytes through the activated endothelium at the site of injury (69, 72, 73).

Taking into account the onset and progression of inflammation, it might be considered as either an acute or a chronic process. Physiological inflammation is frequently self-limited, showing a transient abnormal condition characterized by the production of pro-inflammatory cytokines that is followed closely and in a specific temporal manner by the production of anti-inflammatory cytokines with counter-regulating effects that attenuates or resolve the inflammatory process, thus contributing to the restoration of the homeostasis to the tissue and the eradication of the source of damage (75, 76). This counter-part inflammatory response is mediated mainly by tissue-resident and recruited macrophages (activated monocytes). However, when the local inflammatory stimuli persists or the mechanisms of repair fails, chronic inflammation ensues due to sustained pro-inflammatory mechanisms that may lead to a pathological state, usually seen in chronic infections and autoimmune diseases (69, 74).

Moreover, it can also be the case that the inflammatory response overwhelms local regulatory mechanisms and leads to systemic inflammation that, in turn, might affect metabolism in distant tissues and organs and eventually leads to the pathogenesis of inflammatory syndromes.

Chronic systemic inflammation occurs in a variety of severe diseases including cancer, diabetes, cardiovascular diseases and aging-related neurological diseases, and are associated with an imbalance of tissue homeostasis instead of the typical initiators of inflammation (infection, injury) (77, 78). When the inflammatory response is dysregulated and cannot be attenuated organ dysfunction could occur (70, 74).

The inflammatory response is typically initiated by pattern recognition receptors, as Toll-like receptors (TLRs) and NOD-like receptors (NLRs), that are expressed on the surface of immune cells like neutrophils and macrophages. Upon recognition of pathogen-associated molecular pattern molecules (PAMPs) and damage-associated molecular pattern molecules (DAMPs) (such as lipopolysaccharide (LPS) of Gram-negative bacteria and nucleic acids from viruses, among others), these receptors activate inflammatory signaling pathways (45). The intracellular cascades triggered by TLRs activate downstream kinases like IkB and MAP kinases that regulate different transcription factors such as NF-kB and AP-1, that ultimately induce the expression of pro-inflammatory genes, such as cytokines (IL-1b, IL-6, TNF-a) and chemokines, that serve to recruit additional immune cells, and proteins like iNOS that generate nitric oxide (NO). There are also other types of receptors specialized in detecting intracellular PAMPs and DAMPs, as mentioned above, the NLRs. These receptors are involved in the formation of the multi-protein signaling complexes known as inflammasomes (79). Activation of NLRP3, the most fully characterized inflammasome due to its association with many inflammatory diseases, drives caspase-1 activation and maturation of pro-IL-1b, thus acting as sensors initiating innate inflammatory responses that are triggered by a variety of danger signals including metabolic stress (80–82). High levels of reactive oxygen species (ROS) showed to activate NLRP3 (71).

When the inflammatory response overwhelms local regulatory mechanisms leading to systemic inflammation, metabolism in distant tissues and organs may be affected. Interestingly, these structures might also show different degrees of sensitivity. In this sense, as mitochondria can perceive signals of inflammation is one of the first organelles to be affected by a dysregulation in the systemic inflammatory response and has been associated with the progression of physiopathological mechanisms. Therefore, there is a growing attention in the biology and medical research field related to mitochondrial modulation during inflammatory syndromes.

Besides the mitochondria major functions of synthesized ATP through the process of oxidative phosphorylation, and the other key cellular events through the generation of ROS, as was described above, the organelle also execute other important roles in regulating cellular apoptosis and modulation of calcium metabolism (83) that in turn regulates the metabolic state of the cell through different signaling pathways. Appropriate mitochondrial function is essential to supply energy requirements of immune cells showing an important role in the immunity regulation (84). In this sense, mitochondria bioenergetics is differentially regulated in activated macrophages (M1) and alternatively (M2) macrophages. M1 macrophages are important for clearance of pathogen infections, while M2 are involved in the termination inflammatory phase showing an anti-inflammatory phenotype (82).

There are several mechanisms by which mitochondria may lead to tissue dysfunction: (a) reduction in cellular high energy [understood as adenosine triphosphate (ATP)] levels due to impairment of mitochondrial metabolic pathways, (b) generation of active species, that can damage cell organelles directly (through the reaction with cellular components) or indirectly (by the activation of signaling pathways), (c) involvement in the intrinsic pathway of cellular apoptosis, and (d) impaired Ca^2+^ metabolism, that subsequently triggers an overproduction of reactive oxygen and nitrogen species (85).

The first sign of mitochondrial function lost upon inflammation is an altered oxygen consumption by the electron transport chain, which directly affects the oxidative phosphorylation leading to a decreased capacity to synthesize ATP. Moreover, increased production of ROS by damaged mitochondria could directly activate NLPR3 inflammasome (86) that may work together with NFkB signaling, perpetuating the inflammatory response and consequently conducting to an overstimulation of the inflammatory response (82). Therefore, inflammation induced by oxidative stress acts as a feedback system sustaining a harmful condition that could result in tissue damage and trigger chronic inflammation.

Mitochondria has been also linked to inflammation through another mechanism where mitochondrial DNA (mtDNA) may trigger innate immunity. Various mitochondrial stressors can lead to mtDNA leakage through the mitochondrial outer membrane permeabilization (MOMP) (87). Stressed mitochondria could become a relevant oxidized mtDNA release source (88). Moreover, incomplete degradation of damage mitochondria also causes subsequent cytoplasm accumulation (89). Once mtDNA reach the cytosol is a suitable ligand for the DNA sensing protein cGAS that catalyzes the production of the secondary messenger cGAMP (90–93). Afterwards, cGAMP binds the adaptor molecule STING an endoplasmic reticulum (ER)-resident protein that triggers innate immunity via activation of TBK1 kinase, responsible of IRF3 phosphorylation, the transcription factor initiates type I IFN response (89, 90, 94). The intrinsic function of the cGAS-STING pathway elicits inflammatory diseases regulation. Therefore, is a relevant pathway from the clinical point of view for future translational approach.

Finally, dysregulation of the systemic inflammatory response is associated with the induction of organ dysfunction and multiple organ failure (86, 95). The mechanisms underlying this deleterious effect could be a consequence of mitochondrial impaired bioenergetic processes impacting complex cellular and physiological functions. Unraveling the mechanisms that interconnect mitochondrial dysfunction, metabolism and systemic inflammation would significantly contribute to the better understanding of many chronic inflammatory diseases.

## Mitochondrial Function in Inflammatory and Metabolic Diseases Associated With Air Pollution Exposure

### Airborne PM Exposure Health Outcomes

According to the World Health Organization (WHO), 9 out of 10 people worldwide breathe low-quality air (96). Consequently, more than 9 million premature deaths occur every year due to the joint effects of household and ambient air pollution exposure (97). Recently, it has been estimated that breathing polluted air in urban environments reduces life expectancy by almost 3 years globally (98). In addition, model projections based on business-as-usual emission scenarios suggest that outdoor air pollution contribution to premature mortality could be doubled by 2050 (99). Human can be involuntary exposed to pollutants mainly through tissue or organs that directly interact with PM present in air pollution, as for example the respiratory tract by inhalation, penetration through the skin or eyes and ingestion via the gastrointestinal tract (100). Due to the adverse health effects associated with air pollution, increased incidence of respiratory diseases, such as pneumonia, chronic obstructive pulmonary disease (COPD), and lung cancer, has been observed (101). However, PM exposure on primary organs produce inflammation leading to systemic complications resulting in distant organs defects, such as heart (102, 103). Cardiovascular diseases largely account for the majority of the increase in morbidity and mortality rates (104). In fact, according to the Global Burden of Disease study, air pollution is responsible for one-fourth of the total death count from ischemic heart disease and stroke (105).

### Air Pollution Composition and Classification

Air pollution is comprised of a mixture of gases (such as carbon monoxide, sulfur dioxide, nitrogen oxides, and ozone) and airborne PM (106). Besides the complex nature of air pollution and the coexistence of many compounds that may together contribute to the reported negative health impact, numerous epidemiological studies indicate that PM is the main responsible for the health outcomes (107). PM is a heterogeneous mixture of solid and liquid particles suspended in air that are broadly categorized by their size: PM with an aerodynamic diameter <10 μm (PM_10_) can lodge in the upper respiratory airways and exert a local toxic effect. However, the most harmful particles are those with a diameter <2.5 μm (PM_2.5_), as they can enter deeper into the lung and reach the alveoli (108) or penetrate the skin as hair follicles extend provide a route of penetration for particles from the dermis to the open surface of the skin (109, 110). While coarse particles (PM_10_) usually arise from natural sources, fossil fuel combustion from transport, industry, and power generation mostly account for air pollution fine particles (PM_2.5_) burden in urban environments (106). In addition, it has been suggested that nano-scale particles are even able to break through different epithelia and translocate into the bloodstream (111), being potentially able to induce direct damage to peripheral cells and tissues.

Smaller particles present a higher surface to volume ratio providing a larger area were different compounds could be adsorbed (112). Associations have been stablished between air pollution specific components and the toxic mechanisms elicited. Transition metals within the fine PM fraction which typically include iron (Fe), vanadium (V), nickel (Ni), chromium (Cr), copper (Cu), cadmium (Cd) and zinc (Zn), on the basis of their ability to generate ROS in biological tissues and produce an oxidative stress condition (113). Most of these metals have the ability of participate in Fenton-like reactions and contribute to an increase ROS release, initiating oxidative damage mechanisms (113, 114). Regarding organic compounds, polycyclic aromatic hydrocarbons (PAHs) can also be found coating particles. The cytotoxic mechanisms associated to PAHs involve also involve O_2_-derived free radicals mainly from mitochondria (115).

### PM Detrimental Effects on the Ocular Surface

In those organs constantly exposed to the environment, the epithelia act as the first physical barrier against pollutants becoming more vulnerable (100). For example, it has been shown that PM exacerbates irritation, burning, foreign body sensation, redness, itching in the eyes of people living in urban areas (116–121). Detrimental PM effects on ocular surface are associated with oxidative stress and proinflammatory pathways (122–126). In this sense, both corneal and conjunctival epithelial cells exposed to different PM surrogates *in vitro* increase the inflammatory mediators' production (127–130). In turn, the cytokines release, such as IL-6, IL-8, TNF-α, IL-1β, and MCP-1, lead to morphological changes due to cellular hyperplasia affecting the refractive power of the cornea and the vision process (131, 132). Moreover, the eyes are highly vascularized, accordingly an important PM-induced mediators' source. It has been shown that conjunctival epithelial cells are able to uptake diesel exhaust particles (DEPs) where the polycyclic aromatic hydrocarbons (PAHs) compounds present in the particles, trigger the increased ROS released from mitochondria in early stages, and from NOX, in particular NOX4 later. In agreement with studies presenting a link between IL-6 and NOX expression and vice versa (133–136), DEP-induce NOX4 activation along with a proinflammatory response mediated by IL-6. In addition, ROS production induce a redox imbalance sufficient to initiate nuclear factor erythroid 2-related factor 2 (Nrf2) signaling that translocates to the nucleus to enhance the cellular antioxidant capacity (123). Interestingly, whole-body exposure to urban air models in mice showed similar results with macromolecular oxidative damage due to redox imbalance along with an inflammatory response modulated by the increase in IL-10 levels after 1 week of exposure, which early regulates the release of TNF-α and IL-6 (124).

### Toxic Mechanisms of PM Exposure on the Skin

Skin is one of the main organs exposed to outdoor pollutants because it provides a major interface between the body and the environment. Similar to the ocular surface offers a biological barrier against air PM where the stratum corneum as is the upper layer represent the main PM-target (137). Inflammation in the skin and an altered redox homeostasis has been mentioned as relevant PM-induced mechanisms (110, 138–140) associated with the aggravation of skin diseases, including atopic dermatitis, acne, and psoriasis (141–143). It was reported that in keratinocytes altered ROS release may trigger mitogen-activated protein kinase (MAPK) signaling pathways resulting in the activation of redox-sensitive transcription factors NF-κB and AP-1. Once in the nucleus, those transcription factor promote the transcription of a variety of proinflammatory cytokines, including TNF-α, IL-1a, IL-6, and IL-8. IL-1α and IL-1β in keratinocytes (144). In addition, PM-induce ROS production through the NOX4 activation stimulates NF-κB translocation and increased transcript levels of cytokines (145). Assessment of a 3D skin model exposed to PM showed increased levels of oxidative damage markers resulting in activation of NF-κB, increased levels of proinflammatory marker COX-2, release of IL-1α, and DNA damage (146). Comparable to the mechanisms observed in eye and lung tissue exposed to airborne pollutants, skin can absorb particles induced tissue damage, suggesting a cascade of effects that are driven by inflammatory processes and oxidative damage, leading to systemic inflammation as a consequence (147) and also endothelial dysfunction (148, 149).

### The Gastrointestinal Tract as a PM Target

The oral tracts along as well as the respiratory system and the skin are the common direct routes of exposure to outdoor pollutants. Particles can access the gut as a result of contaminated food ingestion or indirectly by inhalation (150, 151). Therefore, in recent years research aiming to evaluate the gastrointestinal (GI) tract toxic mechanisms after pollutants exposure have increased. Those studies are based on the premise that GI epithelium behave similar than other organs epithelial cells, like skin or lung tissue, in response to PM exposure (100). Thus, PM also triggers increased ROS generation, which initiate the activation of redox sensitive transcription factor NF-κB (152). In the GI cells NF-κB regulates transcription of myosin light chain kinase (MLCK) (153), affecting perijunctional actin, occludin, and ZO1 at tight junctions, resulting in alteration of gut permeability (152, 154). It has also been observed increased plasma levels of proinflammatory TNF-a and MCP-1 due to PM exposure (153). Take all those results into consideration presence of PM in the GI tract increased oxidative stress and inflammation, leading to structural tissue damage which results in mediators leaking. PM-initiated systemic inflammation may worsen several GI tracts issues, including Crohn's disease (155–157), inflammatory bowel disease (IBD) (158), appendicitis (159), and colorectal cancer (160, 161).

### PM Toxicity Within the Brain

Epidemiological studies in humans have shown that high levels of PM are associated with cognitive function changes in children, adults, and the elderly (162). Alterations at the olfactory level, hearing deficits, symptoms of depression and other neuropsychological effects have also been reported (163). The main mechanisms of neurotoxicity produced by PM seem to be related to oxidative stress and neuroinflammation, which are also related to the pathophysiology of various neurodegenerative diseases (164). The way in which the PM reaches the brain and how the particles cause the damage has not been clarified yet. PM that circulates through the nasal compartment can cross the epithelial barrier into the bloodstream or translocate along the axon of the olfactory nerve and reach the central nervous system. Therefore, the adverse effects produced by the inhalation of particles could be the result of a direct effect of the particles via a route from the nasal mucosa to the axon of the olfactory nerve and from there to the olfactory bulb, or by an indirect route through of the systemic inflammatory reaction initiated by increased levels of proinflammatory mediators released into the bloodstream, and oxidative stress (165). Hence, alike GI tract, brain toxicity might begin through direct as well as indirect PM-initiated mechanism.

### Cardiorespiratory Diseases Associated to PM Exposure

Following PM inhalation, the activation of oxidative stress and inflammatory pathways largely account for PM biological effects, both locally as well as systemically and in secondary organs, such as the heart (103, 166). In the lung, increased levels of pro-inflammatory cytokines, including Interleukin (IL)−1β, Tumor Necrosis Factor (TNF) -α, IL-6, and Monocyte Chemoattractant Protein (MCP) −1, are a frequent finding after PM exposure (167). Therefore, lung inflammatory cell recruitment is usually observed following PM exposure, both in humans (168) and in different animal models (169, 170). Increased plasma levels of these inflammatory mediators have been also associated with episodic elevations in PM in large-scale cohort studies (171), indicating that PM exposure triggers an inflammatory response that is not only confined to the lung, but is also systemic. As a result, metabolism is impaired in distant organs, such as in the heart (172).

Alveolar macrophages play a central role in maintaining lung homeostasis through the removal of exogenous materials and microorganisms from the respiratory surface by phagocytosis, including PM (173, 174). Evolution has refined alveolar macrophages ability for the recognition and clearance of pathogens. However, non-biological anthropogenic PM escapes from this machinery and overwhelms cell capacity for foreign material removal, leading to uncontrolled cell activation and ROS production, as well as an exaggerated inflammatory response and pro-inflammatory cytokine release (175, 176). Activation of the NLRP3 inflammasome following PM uptake seems to represent a central step in the cellular inflammatory response to PM in alveolar macrophages (177). Interestingly, PM has been also shown to accumulate inside mitochondria (174), suggesting a specific direct effect of PM over this organelle. Accordingly, PM exposure induces altered mitochondrial ultrastructure in alveolar macrophages, including swelling, cristae disorder, and organelle fragmentation at high doses, as well as modulation of mitochondrial fission/fusion gene expression (178). Moreover, quinones and polycyclic aromatic hydrocarbons (PAHs) in PM seem to drive mitochondrial depolarization and ROS production *in vitro* (179). We and others have recently showed that macrophage depletion by intranasal or intratracheal administration of clodronate liposomes reduce pulmonary pro-inflammatory cytokine release following PM exposure, this preventing enhanced thrombosis (180) as well as aggravated myocardial remodeling following experimental myocardial infarction in PM-exposed mice (181). These findings highlight the role of alveolar macrophages in the oxidative and inflammatory response following PM exposure, which negatively impact cardiorespiratory disease onset and progression. Moreover, the role of PM-induced mitochondrial ROS in the release of IL-6 and other pro-inflammatory cytokines in alveolar macrophages was confirmed by treatment with Metformin a complex I inhibitor which reduce mitochondrial complex III ROS production (182). Therefore, diminished mitochondrial ROS release might reduce the risk of PM-induced thrombosis (183).

**Figure 3:**
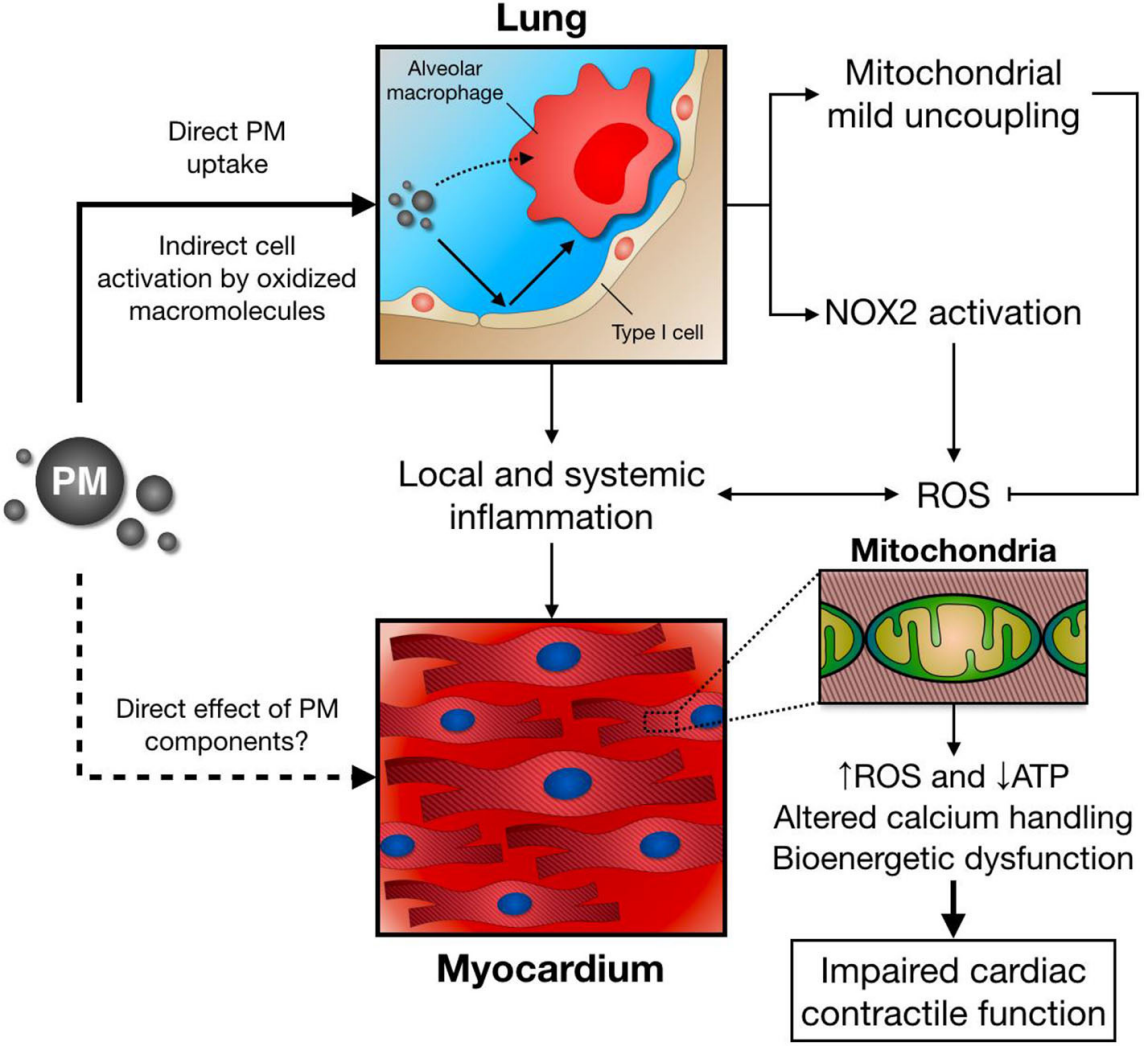
Following PM inhalation, direct and indirect mechanisms induce alveolar macrophage activation and pro-inflammatory cytokine release. In parallel, lung NOX2 activation leads to increased ROS production, while mitochondrial mild uncoupling might ameliorate excessive ROS release. Together with local and systemic inflammation, and potential direct effects of PM/PM components, this scenario has a negative impact over distant organs, such as the heart. As a consequence, cardiomyocyte mitochondrial function, energy metabolism, and calcium handling are impaired, leading to deficient contractile function after PM exposure.

We and others have also studied the role of the exposure to air pollution PM over lung and heart redox metabolism (184, 185), in which altered mitochondrial respiration together with enhanced NOX2 activity plays a central role as shown in Figure 3 (186, 187). Interestingly, NOX2 seems to account for increased ROS production in the lung following PM exposure, while mitochondrial mild uncoupling, characterized as increased oxygen consumption rate and decreased inner membrane potential, together with decreased ATP production rate and lower efficiency of the oxidative phosphorylation process (lower P/O ratio), may prevent further ROS release from this organelle (186). When increasing electron transport rate at the respiratory chain complexes, mitochondrial ROS production is attenuated by different mechanisms: First, mitochondria can significantly reduce ${\text{O}}_{2}^{.-}$ production by decreasing oxygen tension in the mitochondrial microenvironment; Second, by favoring more oxidized levels of respiratory chain intermediates; Third, by lowering NADH levels that could be used by mitochondrial matrix flavoenzymes; Forth, by preventing reverse electron transfer due to lower membrane potential (188).

Impaired cardiac mitochondrial function also arises as a central feature of air pollution PM toxicology. Mechanistically, an acute exposure to PM induces a decrease in active, but not rest, state oxygen consumption rate, together with inner membrane depolarization and reduced mitochondrial ATP production (96). Consequently, deficient contractile and lusitropic reserve is observed in PM-exposed mice, as the heart fails to properly increase cardiac contractility after a β-adrenergic stimulus with isoproterenol (187). Blunted mitochondrial ATP supply in mice breathing PM may account for this effect, as decreased ATP levels are a frequent finding in the failing heart (189, 190). Interestingly, this cardiac mitochondrial bioenergetic dysfunction seems to be partially mediated by an inflammatory response triggered by PM exposure, since impaired mitochondrial respiration and cardiac contractility is attenuated by pretreatment with a chimeric anti-TNF-α antibody (Infliximab) in PM-exposed mice (172).

Given that heart perfusion with a low-calcium Krebs buffer also prevents impaired contractility in PM-exposed mice (172), calcium overload may also contribute with this scenario. In fact, altered calcium homeostasis has been recently reported in cardiomyocytes incubated with PM (191), which might be explained by hampered mitochondrial calcium uptake by aromatic chemicals in PM (179) and decreased activity of sarco/endoplasmic reticulum calcium-ATPase 2a (SERCA2a), a major regulator of cytosolic calcium concentration that couples ATP hydrolysis with calcium transport into the sarcoplasmic reticulum during relaxation (192). Moreover, as PM exposure impairs cardiac mitochondrial respiration, deficient mitochondrial ATP supply in PM-exposed mice heart represent a plausible link between ROS production, altered calcium handling, and impaired myocardial contractile function and relaxation. In support of this concept, sarcomere shortening in cardiomyocytes incubated with PM was prevented by the mitochondrial targeted antioxidants Tiron and MitoTEMPOL (191).

Recently, updated epidemiological studies found a positive correlation between air pollution exposure and type 2 diabetes mellitus incidence (193) and mortality (194). Therefore, the role of air pollution PM exposure over metabolism, adipose tissue inflammation, and obesity gained crescent attention. Mechanistically, the exposure to PM in a mice model of diet induced obesity has been shown to aggravate insulin resistance and inflammation in white adipose tissue (195). Moreover, PM exposure induced increased ROS production and downregulation of uncoupling protein (UCP) −1 in brown adipose tissue (196). UCP-1 is a mitochondrial inner membrane protein that dissipates membrane potential to produce heat, and therefore modulates thermogenesis and protects against excessive ROS production (197). It remains unclear whether adipose tissue inflammation and altered mitochondrial function represent a cause or consequence of altered metabolism following PM exposure. In this context, the precise role of mitochondrial respiration, ROS release, and heat production needs to be further addressed in future studies.

Taken together, impaired mitochondrial respiration, enhanced ROS release, and deficient ATP supply (and maybe also uncoupling), play a central role in the adverse health effects reported after air pollution PM exposure. In this context, the modulation of mitochondrial function (e.g. by mitochondrial targeted antioxidants) arises as a potential therapeutic target to prevent excessive lung inflammatory response, as well as impaired cardiac contractility and metabolism in PM-exposed individuals at particular high risk.

## Conclusions

The mitochondria-dependent mechanisms associated with inflammation are still poorly understood. A better understanding of the cellular pathways underlying this phenomenon would allow a more targeted approach to face the adverse effects linked to inflammatory syndromes. An interplay between mitochondria and NOX as specific ROS sources in inflammation has been recognized, where the redox crosstalk stimulates each other in a positive feedback fashion, resulting in a complex oxidative stress and redox signaling network. As a case study, inflammation and altered mitochondrial function represent a relevant mechanism of altered cell metabolism following PM exposure may contribute to the increased morbidity and mortality associated with polluted areas. The modulation of mitochondrial function by mitochondrial targeted antioxidants arises as a potential therapeutic target to prevent an excessive inflammatory response.

## Author Contributions

All authors listed have made a substantial, direct and intellectual contribution to the work, and approved it for publication.

## Conflict of Interest

The authors declare that the research was conducted in the absence of any commercial or financial relationships that could be construed as a potential conflict of interest.
